# Oestrogen receptor alpha in pulmonary hypertension

**DOI:** 10.1093/cvr/cvv106

**Published:** 2015-03-12

**Authors:** Audrey F. Wright, Marie-Ann Ewart, Kirsty Mair, Margaret Nilsen, Yvonne Dempsie, Lynn Loughlin, Margaret R. Maclean

**Affiliations:** 1 College of Medical, Veterinary, and Life Sciences, Research Institute of Cardiovascular and Medical Sciences, University of Glasgow, Room 448, West Medical Building/Wolfson Link Building, Glasgow G12 8QQ, UK; 2 School of Health and Life Sciences, Glasgow Caledonian University, Glasgow G4 0BA

**Keywords:** Pulmonary hypertension, Oestrogen, Oestrogen receptor alpha, Serotonin, BMPR2

## Abstract

**Aims:**

Pulmonary arterial hypertension (PAH) occurs more frequently in women with mutations in bone morphogenetic protein receptor type 2 (BMPR2) and dysfunctional BMPR2 signalling underpinning heritable PAH. We have previously shown that serotonin can uncover a pulmonary hypertensive phenotype in BMPR2^+/−^ mice and that oestrogen can increase serotinergic signalling in human pulmonary arterial smooth muscle cells (hPASMCs). Hence, here we wished to characterize the expression of oestrogen receptors (ERs) in male and female human pulmonary arteries and have examined the influence of oestrogen and serotonin on BMPR2 and ERα expression.

**Methods and results:**

By immunohistochemistry, we showed that ERα, ERβ, and G-protein-coupled receptors are expressed in human pulmonary arteries localizing mainly to the smooth muscle layer which also expresses the serotonin transporter (SERT). Protein expression of ERα protein was higher in female PAH patient hPASMCs compared with male and serotonin also increased the expression of ERα. 17β-estradiol induced proliferation of hPASMCs via ERα activation and this engaged mitogen-activated protein kinase and Akt signalling. Female mice over-expressing SERT (SERT^+^ mice) develop PH and the ERα antagonist MPP attenuated the development of PH in normoxic and hypoxic female SERT^+^ mice. The therapeutic effects of MPP were accompanied by increased expression of BMPR2 in mouse lung.

**Conclusion:**

ERα is highly expressed in female hPASMCs from PAH patients and mediates oestrogen-induced proliferation of hPASMCs via mitogen-activated protein kinase and Akt signalling. Serotonin can increase ERα expression in hPASMCs and antagonism of ERα reverses serotonin-dependent PH in the mouse and increases BMPR2 expression.

## 1. Introduction

The incidence of pulmonary arterial hypertension (PAH) is greater in females. For example, the female-to-male ratio is currently reported in the REVEAL Registry as approximately 4.1:1 for idiopathic PAH (IPAH) and 3.8:1 for associated PAH (APAH).^1,2^ Dysfunctional bone morphogenetic protein receptor 2 (BMPR2) signalling is recognized to play a pivotal role in the development of PAH and mutations in BMPR2 are responsible for ∼80% of heritable PAH (HPAH) cases.^3^

Female gender is also known to increase the penetrance of BMPR2 mutations in HPAH.^3^ Reasons for these gender differences remain unclear, however, there is converging evidence suggesting that sex hormones, in particular oestrogens, are a major risk factor in females with PAH and play a pivotal role in PAH pathogenesis. Clinically, polymorphisms in aromatase (CYP19A1), the enzyme that synthesizes oestrogen, are associated with higher oestrogen levels and an increased risk of PH development in female patients with advanced liver disease.^4^ In line with this, physiological concentrations of oestrogen mediate proliferation of human PASMCs and an inhibitor of endogenous oestrogen synthesis can reverse the development of PH in female rodents.^5,6^

We have previously demonstrated that serotonin can uncover a PH phenotype in BMPR2^+/−^ mice via decreased BMPR2 signalling.^7^ Multiple studies have implicated serotonin and the serotonin transporter (SERT) in the pathogenesis of PAH. SERT overexpression and/or activity are observed in pulmonary arteries and lungs from patients with PAH and are associated with an exaggerated proliferative response.^8^ In serotonin-dependent rodent models of pulmonary hypertension (PH), including mice over-expressing the calcium-binding protein S100A4 (Mts1), dexfenfluramine-treated mice and mice over-expressing the SERT^+^, only the female mice develop PH.^9–11^ We have also previously demonstrated that 17β-estradiol plays a key role in the development of PAH in female SERT^+^ mice.^6^ For example, ovariectomized female SERT^+^ mice do not develop PAH, while re-introduction of 17β-estradiol completely re-establishes the disease phenotype.^6^ At the cellular level, 17β-estradiol can up-regulate SERT expression and proliferation in human pulmonary arterial smooth muscle cells (hPASMCs).^6^

The effects of oestrogen are primarily mediated by the activation of three oestrogen receptors (ERs), ERα, ERβ, and G-protein-coupled receptor (GPER).^12^ ERα and ERβ mediate both genomic and non-genomic oestrogen signalling, while very rapid non-genomic effects of oestrogen have been attributed to the GPER. Experimentally administered *exogenous* oestrogen can protect against hypoxic PH in intact male rats and this is mediated by ERα and ERβ.^13^ However, we have recently demonstrated that hPASMCs synthesize oestrogen endogenously via aromatase expression and this expression is increased in female hPASMCs.^5^ This *endogenous* oestrogen plays a pathogenic role in the development of PH in female rats and mice and this may be via decreased BMPR2 expression. Indeed, inhibition of ERα reverses PH in female hypoxic mice while having no effect in male hypoxic mice.^5^

The aims of this study were therefore to characterize the expression of ERs in human lung and PASMCs and to examine the role of endogenous oestrogen, via ERα activation, in a serotonin and oestrogen-dependent mouse model, the female SERT^+^ mouse.

## 2. Methods

### 2.1 Ethical information

All experimental procedures conform with the United Kingdom Animal Procedures Act (1986) and with the ‘Guide for the Care and Use of Laboratory Animals’ published by the US National Institutes of Health (NIH publication No. 85-23, revised 2011), and ethical approval was also granted by the University of Glasgow Ethics Committee. Experimental procedures utilizing human pulmonary artery smooth muscle cells (PASMCs) conformed with the principles outlined in the Declaration of Helsinki. Informed consent was given for the use of cells. Studies were approved by Cambridgeshire 1 Research Ethics committee (REC reference: 08/H0304/56).

### 2.2 Generation of genetically modified SERT^+^ mice

Mice over-expressing the human SERT gene transcript were generated and supplied by Professor Tony Harmer, University of Edinburgh, UK. SERT^+^ mice were generated using the C57BL/6×CBA wild-type strain. See Supplementary material online for more details.

### 2.3 *In vivo* effects of MPP dihydrochloride administration

Two days prior to the induction of chronic hypoxia, SERT^+^ mice and/or control littermates were administered with slow release pellets containing either ERα antagonist, MPP dihydrochloride [chemical name- 1,3-Bis(4-hydroxyphenyl)-4-methyl-5-[4-(2-piperidinylethoxy)phenol]-1H-pyrazole dihydrochloride] (MPP) 2 mg kg^−1^ day^−1^, or vehicle. See Supplementary material online for more details.

### 2.4 Assessment of PH *in vivo* and vascular remodelling assessment

All haemodynamic measurements were carried out under general anaesthesia using 1–2% (v/v) isoflurane supplemented with O_2_. Right-ventricular systolic pressure (RVSP) was measured by a transdiaphragmatic approach by advancing a heparinized needle into the mid-portion of the abdomen using a micromanipulator.^14^

Systemic arterial pressure (SAP) was obtained by cannulation of the left common carotid artery as previously described.^14^ Right-ventricular hypertrophy (RVH) was assessed as a ratio of the weight of the right ventricle (RV) over the weight of the free left ventricle plus septum (LV+S). Haemodynamic assessment was carried out in 6–12 mice for each group. Animals were randomly allocated to groups and all measurements, assessments, and analysis carried out in a blinded fashion. See Supplementary material online for more details

### 2.5 Vascular remodelling assessment

Pulmonary vascular remodelling was assessed in lung sections stained with alpha-smooth muscle actin and microscopically examined. See Supplementary material online for more details.

### 2.6 Oestrogen receptor and SERT immunolocalization in human lung

Briefly, 5 µm sagittal sections of fixed human lung were deparifinized and rehydrated as discussed earlier. After epitope retrieval, oestrogen receptor (ER) alpha (ERα) (Santa Cruz, sc-7207; 1 µg/mL), ERβ (Abcam, ab-3577; 5 µg/mL), GPER (Abcam, ab-39742; 5 µg/mL), and SERT (Abcam, ab-44520: 1:200) were incubated overnight at 4°C. Lung sections were counterstained with haematoxylin. Distribution was assessed by staining consecutive sections with alpha smooth muscle actin (for medial cells) and von-Williebrand factor (for endothelial cells).

### 2.7 Human PASMC proliferation

Proliferation in hPASMCs was assessed by measuring DNA synthesis by [^3^H] thymidine incorporation^15^ in the presence of agonists for ERα, ERβ, and GPER [4,4′,4″-(4-Propyl-[1*H*]-pyrazole-1,3,5-triyl)*tris*phenol (PPT), Diarylpropionitrile or 2,3-*bis*(4-Hydroxyphenyl)-propionitrile (DPN) & (±)-1-[(3aR*,4S*,9bS*)-4-(6-Bromo-1,3-benzodioxol-5-yl)-3a,4,5,9b-tetrahydro-3 *H*-cyclopenta[*c*]quinolin-8 -yl]-ethanone (G1), respectively] (0.01–10 nmol/L) and 1 µM antagonists [ERα: MPP, ERβ: 4-[2-Phenyl-5,7-*bis*(trifluoromethyl)pyrazolo[1,5-*a*]pyrimidin-3-yl]phenol (PHTPP), or GPER: (3aS*,4R*,9bR*)-4-(6-Bromo-1,3-benzodioxol-5-yl)-3a,4,5,9b-3*H*-cyclopenta [*c*]quinolone (G15)] where appropriate. See Supplementary material online for more details.

### 2.8 Immunoblotting

Protein expression was assessed by western blotting in human PASMC lysates and mouse pulmonary artery prepared as previously described^9^ and in more detail in the Supplementary material online.

### 2.9 Quantitative reverse transcription–polymerase chain reaction (qRT-PCR)

Total RNA from mouse tissues and human cells were obtained using the QIAGEN RNeasy mini-kit (Qiagen, Manchester, UK) following the manufacturer's instructions.

Treatment with DNAse 1 (Qiagen) eliminated genomic DNA contamination prior to quantification using a NanoDrop ND-1000 Spectrophotometer (Nano-Drop Technologies, Wilmington, DE, USA). High-capacity cDNA Reverse Transcription kits (Life technologies, Paisley, UK) were used for synthesis of cDNA from total RNA.

A mastermix containing dNTPs, random hexamers, and RNase inhibitor, supplied in the kit was added to total RNA and the following cycling conditions were used to synthesize cDNA: 10 min at 25°C, 30 min at 48°C, 5 min at 95°C and 12°C forever. Quantitative real-time PCR (qRT-PCR) was used to validate mRNA expression using TaqMan® Gene Expression probes (Life Technologies, Paisley, UK), as previously described. See Supplementary material online, *Table S3* for details of assay IDs.

### 2.10 Statistics

Statistical analysis was performed using GraphPad Prism 6 Software. Data were analysed using a two-way ANOVA followed by a Bonferroni's *post-hoc* test, one-way ANOVA followed by a Tukey's or Dunnet's *post-hoc* test, or an unpaired *t*-test where appropriate. Data are expressed as ±SEM.

## 3. Results

### 3.1 Expression of oestrogen receptors and SERT in human lung and PASMCs

**Figure 1:**
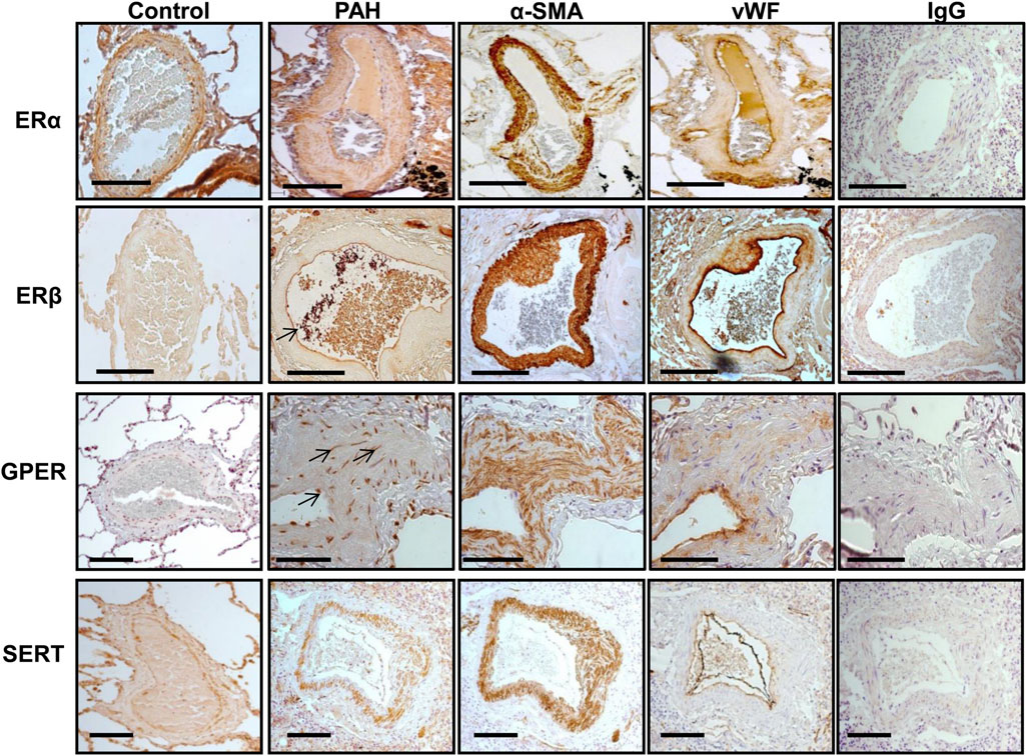
Estrogen receptor and SERT expression profile in human lung. Localization was confirmed by staining of consecutive sections with α-smooth muscle actin (α-SMA) and Von Willebrand (vWB) for smooth muscle and endothelial cells, respectively. ERα was mainly localized to smooth muscle in the pulmonary arteries from control and PAH patients. ERβ expression was largely endothelial (indicated with arrow). GPER staining was punctate in nature and localized to a few endothelial and smooth muscle cells (indicated with arrow). SERT expression is localized to the smooth muscle in patient pulmonary arteries. IgG, immunoglobulin control. Scale bar (−)=200 µm. See Supplementary material online, *Table S1* for patient details.

ER localization was investigated in human lung by immunohistochemistry. ERα, ERβ, and GPER expressions were prominent in pulmonary arteries in both control and PAH patients (*Figure 1*). In PAH patients, ERα was localized to adventitia, smooth muscle cells, and endothelial cells. While some ERβ expression was observed in the adventitia and smooth muscle cells, expression was largely endothelial. GPER was also localized to vascular smooth muscle. The SERT is clearly expressed in the medial layer from patients with PAH. Localization was confirmed by staining of consecutive sections with α-smooth muscle actin (α-SMA) and Von Willebrand for smooth muscle and endothelial cells, respectively (*Figure 1*).

**Figure 2:**
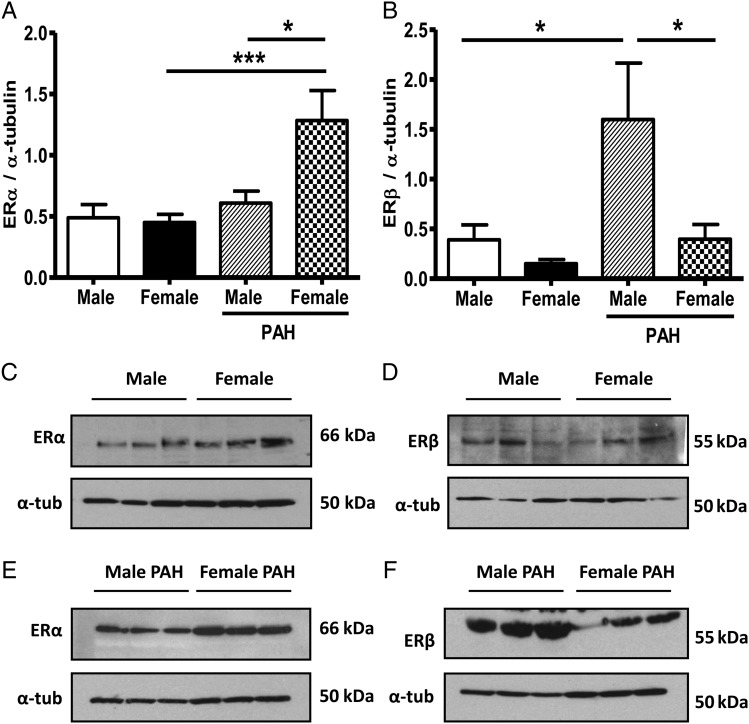
Estrogen-receptor expression is altered between male and female pulmonary artery smooth muscle cells (PASMCs) from PAH patients. ERα and ERβ protein expression in male (*n* = 5) and female (*n* = 6) PASMCs (*A* and *B*). Representative blots are shown (*C*–*F*). Patient information detailed in Supplementary material online, *Table S2*. Control PASMCs: female 1–6, male 1–5; PAH PASMCs: female 1–3, male 1–3, repeated in triplicate. Quantitative data are shown as mean ± SEM and analysed using two-way ANOVA followed by Bonferroni's post -test. *P < 0.05, ***P < 0.001.

ER expression was further investigated in male and female hPASMCs. Levels of ERα and ERβ protein were not significantly different in control female and male hPASMCs (*Figure 2A*–*D*). However, in hPASMCs from PAH patients, ERα protein was expressed at significantly higher levels in female hPASMCs relative to males (*Figure 2A* and *E*) and female controls. Conversely, ERβ expression was significantly less in female hPASMCs compared with males in PAH (*Figure 2B* and *F*). ERβ expression was also significantly higher in male PAH cells compared with male control cells.

### 3.2 Estrogen receptor-α and -β expression in female SERT^+^ mouse lung

**Figure 3:**
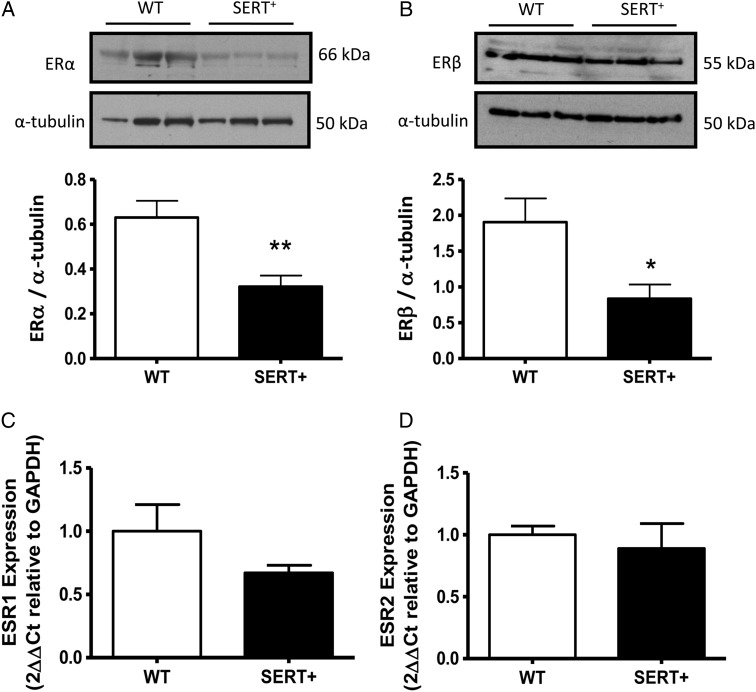
Estrogen-receptor expression is reduced in female SERT^+^ mouse pulmonary artery. ERα and ERβ protein expression in pulmonary artery from female SERT^+^ mice relative to controls (*A* and *B*). ESR1 mRNA transcript in whole lung (*C* and *D*). Representative blots are shown for ERα (*n* = 12 pulmonary arteries from 12 animals) and ERβ (*n* = 9 pulmonary arteries from nine animals) (*A* and *B*). All westerns repeated in triplicate; qRT–PCR, *n* = 9–12 lungs/group. Quantitative data are shown as ±SEM and analysed using an unpaired *t*-test. *P < 0.05, *P < 0.01 vs. wild-type (WT).

ER expression was also investigated in the pulmonary vasculature and lungs from female SERT^+^ mice that develop spontaneous PH in normoxic conditions. In pulmonary arteries from female SERT^+^ mice, there was reduced ERα and ERβ protein levels relative to wild-type control mice (*Figure 3A* and *B*); the mRNA levels of ESR1 and ESR2, the genes encoding ERα and ERβ, were, however, found to be unchanged in preparations from whole lung (*Figure 3C* and *D*).

### 3.3 Effect of ERα antagonism in the SERT^+^ female susceptible *in vivo* mouse model

**Figure 4:**
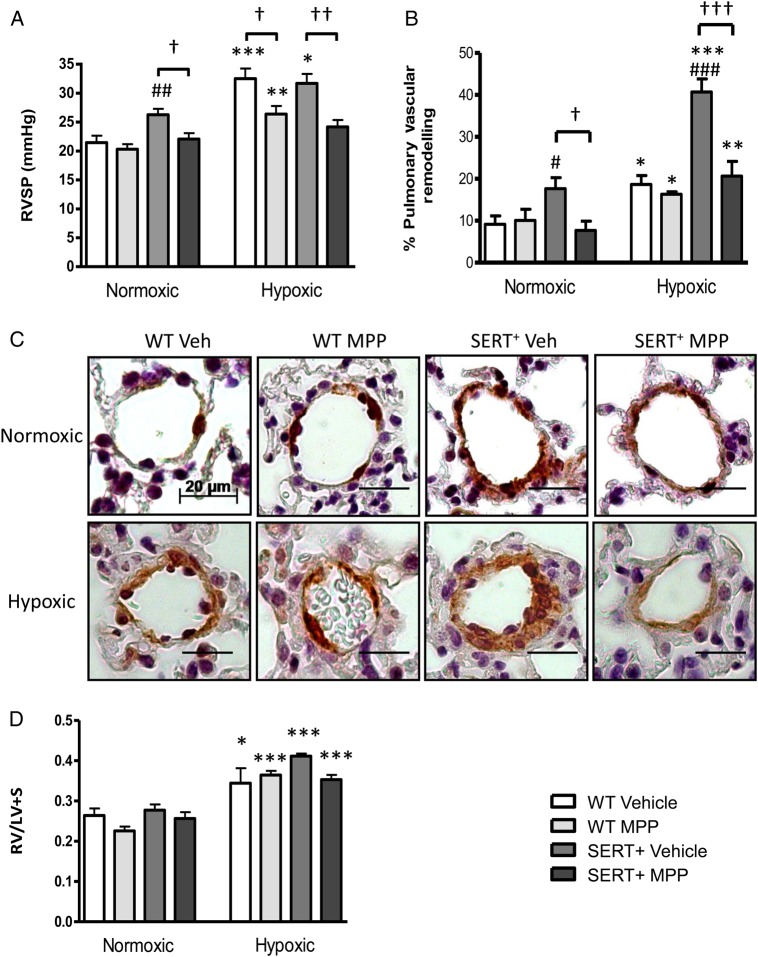
MPP 2 mg kg^−1^ day^−1^ attenuates the development of pulmonary hypertension in female SERT^+^ mice. Effect of MPP on right-ventricular systolic pressure (RVSP) in SERT^+^, hypoxic control, and hypoxic SERT^+^ mice (*A*). Effect of MPP on pulmonary vascular remodelling in normoxic and hypoxic SERT^+^ mouse lungs (*B* and *C*). Effect of MPP on right-ventricular hypertrophy (RV/LV+S) in hypoxic mice (*D*). Representative images from distal pulmonary arteries in each group are shown (alpha-smooth muscle actin stains dark brown, *C*). Data are expressed as ± SEM and analysed using two-way ANOVA followed by a Bonferroni *post-hoc t*-test. *P < 0.05, **P < 0.01, ***P < 0.001 vs. normoxic mice. ^†^*P* < 0.05, ^††^*P* < 0.01, ^†††^*P* < 0.001 vs. vehicle dosed mice. ^#^*P* < 0.05, ^##^*P* < 0.01, ^###^*P* < 0.001 vs. wild-type mice. *n* = 6–11 per group. WT, wild-type. Scale bar (−) = 20 µm.

We wished to investigate if ERα influenced the development of PH in a model that we know to demonstrate female susceptibility. We have previously shown that normoxic female SERT^+^ mice develop spontaneous PAH at 5 months of age in an estrogen-dependent manner, while male SERT^+^ mice do not.^11,16^ In the SERT^+^ mice, the increase in RVSP and pulmonary vascular remodelling was abolished by MPP (*Figure 4A*–*C*). We also exposed these animals to hypoxia, where hypoxic wild-type mice developed PH demonstrating increased RVSP and this was reduced by MPP (*Figure 4A*–*C*). In vehicle-treated SERT^+^ mice, hypoxia caused enhanced pulmonary vascular remodelling relative to hypoxic wild-type vehicle-treated mice and this was also markedly reduced by MPP (*Figure 4B* and *C*). The augmented elevations in RVSP were reversed by MPP administration (*Figure 4A*) while RVH was unaffected (*Figure 4D*). Mean systemic arterial pressure (mSAP) was unaffected by MPP treatment (see Supplementary material online, *Figure S1**A*).

### 3.4 Effects of ERα antagonism on the BMPR2 pathway in female SERT^+^ mice

**Figure 5:**
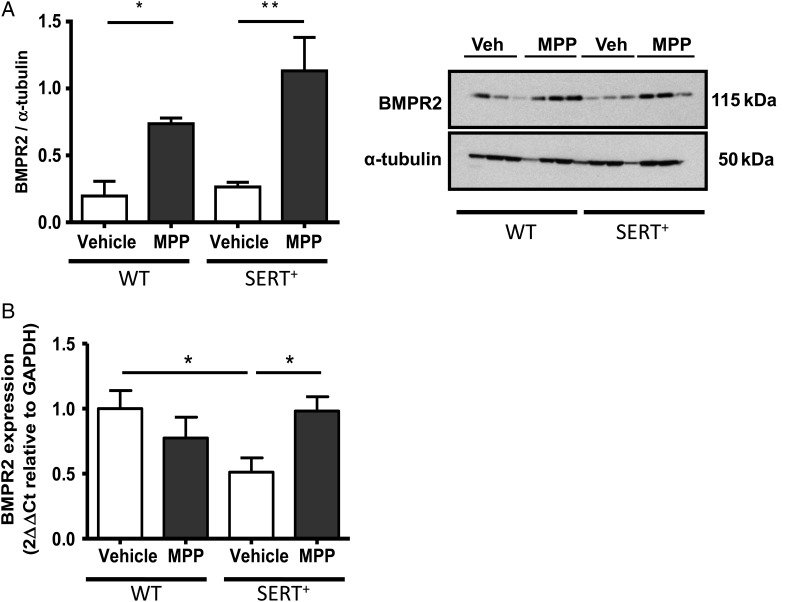
Regulation of the BMPR2 pathway via ERα in female SERT^+^ mouse lung. Effect of MPP on BMPR2 protein in WT and SERT^+^ female lung (*A*). Effect of MPP on BMPR2 mRNA transcript (*B*) in SERT^+^ mouse lung. Quantitative data are expressed as ± SEM and analysed by a two-way ANOVA followed by a Bonferroni post-hoc t-test. *P < 0.05, **P < 0.05 *n* = 6 lungs/group performed in triplicate.

To determine a mechanism by which the estrogen/ERα interaction promotes the pathogenesis of PH in female SERT^+^ mice, we investigated BMPR2 expression in the animals treated with MPP. We examined protein levels of BMPR2 in normoxic female SERT^+^ mice. Although BMPR2 protein levels were unchanged between wild-type control mice and SERT^+^ mice (*Figure 5A*), mRNA transcript was significantly reduced in vehicle-treated SERT^+^ females compared with wild-type controls (*Figure 5B*). Treatment with the ERα antagonist MPP resulted in elevated protein levels of BMPR2 in wild-type mice (*Figure 5A*) and both BMPR2 protein and mRNA transcript levels were increased by MPP in SERT^+^ mice (*Figure 5A* and *B*).

### 3.5 Effects of ER agonists and antagonists in human PASMCs *in vitro*

Our results demonstrated that ERα plays a role in the development of PH in the female SERT ^+^ mouse model. In addition, we demonstrate there are elevated expression levels of ERα in PASMCs from female PAH patients. From these results, we hypothesized that activation of ERα by endogenous oestrogen may be promoting PH especially in females. To determine potential mechanisms by which ERα activation could be facilitating changes in the pulmonary artery in PAH, we examined the effects of oestrogen in female human PASMCs.

**Figure 6:**
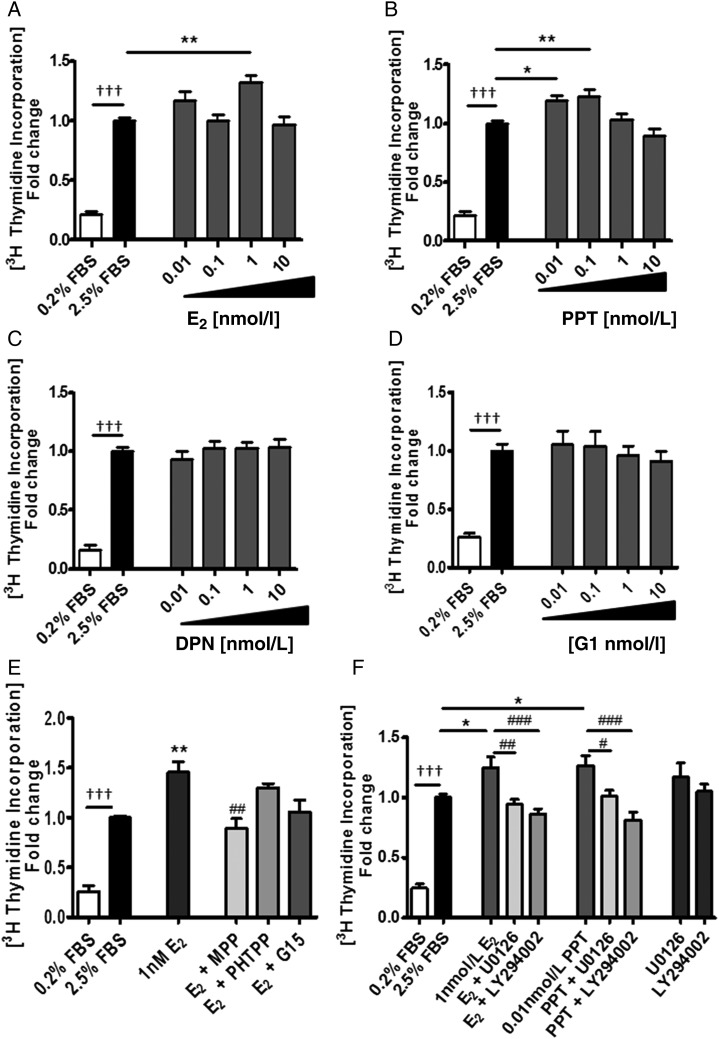
Estrogen induces proliferation of female human PASMCs through ERα in a PI3K/Akt and ERK MAPK-dependent manner. Effect of 17β-estradiol (*A*) and PPT, an ERα agonist (*B*), DPN, an ERβ agonist (*C*) and G1, a GPER agonist (*D*) on proliferation of PASMCs. Effect of the ERα antagonist, MPP (1 µM), an ERβ selective antagonist PHTPP (1 µM) and GPER selective antagonist G15 (1 µM) on E2-induced proliferation of PASMCs (*E*). Effect of U0126 (1 µM), a MEK inhibitor and LY294002 (1 µM), a PI3 K/Akt inhibitoron E2 (1 nM) and PPT (0.01 nM) induced proliferation of PASMCs (*F*). Data are expressed as mean ± SEM and analysed using a one-way ANOVA followed by a Tukey's *post-hoc* test. ^†††^*P* < 0.001 vs. 0.2% FBS; *P < 0.05, **P < 0.01 vs. 2.5% FBS; ^#^*P* < 0.05, ^##^*P* < 0.01, ^###^*P* < 0.001 vs. E2/PPT. *n* = 4 per experiment and performed in triplicate in separate female cell lines (Control PASMCs 1, 2, 3, and 5) depicted in Supplementary material online, *Table S2* (passages 3–5).

**Figure 7:**
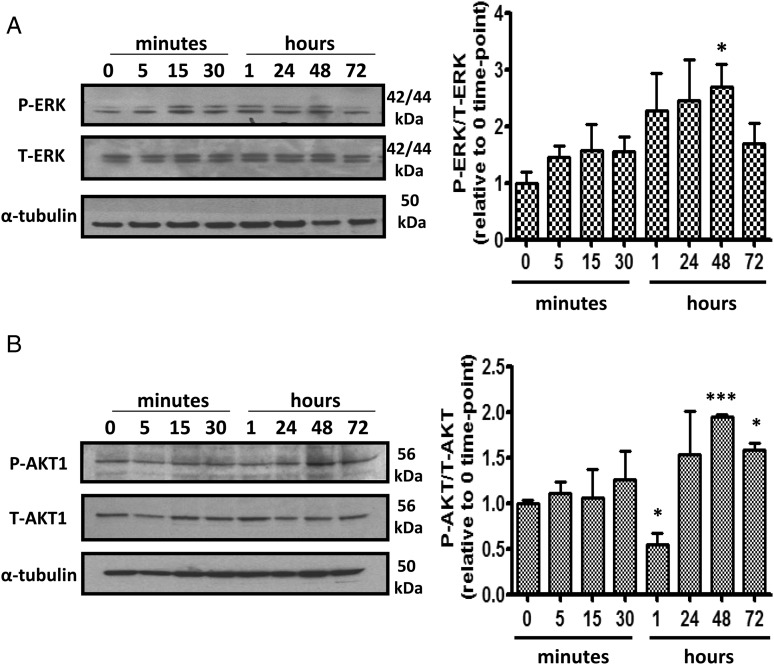
Stimulation of female human PASMCs with 17β-estradiol increases AKT phosphorylation. Effect of 1 nM 17β-estradiol on phosphorylation of ERK-1/2 (*A*) and AKT-1 (*B*) in PASMCs. Representative blots are shown. *n* = 3, *P < 0.05 and ***P < 0.001 vs. 0 time-point control.

The major circulating oestrogen 17β-estradiol was examined at physiological concentrations (0.1–1 nmol/L) and at a supraphysiological concentration of 10 nmol/L. At 1 nmol/L oestrogen-induced proliferation (*Figure 6A*). The ERα selective agonist, PPT, also caused proliferation of PASMCs at 0.01–0.1 nmol/L (*Figure 6B*), whereas diarylpropionitrile (DPN), the ERβ selective agonist and G1, the GPER selective agonist had no proliferative effect (*Figure 6C* and *D*). 17β-estradiol-induced proliferation at 1 nmol/L was inhibited by the ERα selective antagonist MPP (1 µmol/L) while the ERβ selective antagonist (PHTPP, 1 µmol/L) and the GPER selective antagonist (G15,1 µmol/L) had no effect (*Figure 6E*). Akt and MAPKs can phosphorylate and activate ERs and their co-regulators to enhance nuclear transcription and regulate cell survival and proliferation.^17^ We therefore examined the proliferative effects of 17β-estradiol and PPT on PASMC proliferation in the presence of LY294002, a PI3K inhibitor, and U0126, a MAPK/ERK kinase (MEK) inhibitor. Proliferative responses to 17β-estradiol were inhibited in the presence of U0126 and LY294002 (*Figure 6F*). We have also demonstrated that phosphorylation of MAPK/ERK and AKT-1 is increased in response to 1 nmol/L 17β-estradiol (*Figure 7A* and *B*).

### 3.6 Effect of serotonin on ER expression in human PASMCs

We have previously demonstrated that oestrogen can increase the expression of tryptophan hydroxylase 1 (TPH1; the rate limiting enzyme in peripheral serotonin synthesis), SERT, and the 5-HT1B receptor.^6^ Here we wished to examine the effects of serotonin on ER expression in human PASMCs.

In female PASMCs, we demonstrated that expression of ERα protein was significantly increased (see Supplementary material online, *Figure S2**A*) while expression of ERβ was significantly decreased by 1 µmol/L serotonin applied for 24 h (see Supplementary material online, *Figure S2**B*).

## 4. Discussion

This study aimed to investigate the interaction of the ER, gender, and serotonin in the development of PH. First, we investigated the classical ERs, ERα, and ERβ, which predominantly act as transcription factors regulating gene expression, and the novel G-protein-coupled receptor, GPER, which mediates rapid, non-genomic effects of oestrogen.^12^ ERα expression was mainly localized to smooth muscle in the pulmonary arteries from control and PAH patients. Likewise, SERT expression is localized to the smooth muscle in patient pulmonary arteries. ERβ expression was largely endothelial. GPER expression was observed in some endothelial and smooth muscle cells. In humans, expression of ERα and ERβ has previously been identified in endothelial cells and vascular smooth muscle cells of aortic and coronary vasculature as well as in cardiomyocytes.^18,19^ Thus the presence of both ERα and ERβ mediate physiologically important effects of oestrogen in the human vasculature.

Characterization of GPER is less well defined, although enhanced GPER expression has recently been attributed to a pathological role in lung cancer cells.^20^

We hypothesized that ERα drives the PAH phenotype in females. Other studies have also suggested ERα activation plays a role in PAH. For instance, gene expression data from one patient cohort demonstrated an up-regulation of ESR1 in human PAH subjects relative to controls.^21^ Polymorphisms in ESR1 have also been associated with an increased risk of developing portopulmonary hypertension independent of gender.^4^ Uniquely, we report here that ERα expression is markedly increased in human PASMCs from female PAH patients compared with male, while ERβ expression is greatest in PASMCs from male PAH patients. On the other hand, levels of ERα are unchanged between male and female control hPASMCs. We therefore suggest the onset of PAH has a regulatory effect on ERα in pulmonary arteries. In addition, it is known that ERβ inhibits ERα-mediated gene transcription in the presence of ERα in mice and it is possible that such a relationship may influence ER signalling in the pulmonary vasculature.^22^ Curiously, ERβ expression was higher in human PASMCs from male PAH patients compared with male controls. As we show that, *in situ*, ERβ expression may be mainly endothelial, it is unclear if this would have pathophysiological significance.

One limitation of our expression studies in this investigation is the small number of isolated human PASMCs available due to the rare nature of PAH. However, gender differences in tissue localization of ERs during development and disease has been reported in other conditions. In aortic vasculature during aneurysm, a correlation between increased ERα expression in females but not males has been identified and gender differences in vascular function has been attributed to differences in expression, distribution and/or activity of ERs in response to vasoconstrictors.^23–25^ In the lungs of female mice, both ERα and ERβ are required for the full functional and morphological development of alveoli structures, although they have a much smaller effect on alveolar dimensions in male mice.^26,27^ It is likely then, that ERs contribute more significantly to the pathophysiology of PAH in female lung compared with male.

As we are interested in the interactions between serotonin and oestrogen, we investigated if inhibition of ERα with MPP would reverse PH in our female SERT^+^ mouse model. We were also interested in determining if MPP could reverse the additional effects of hypoxia in the SERT^+^ mouse. Our results demonstrate that the ERα antagonist MPP did reverse PH in both the normoxic and hypoxic SERT^+^ mouse. This suggests that activation of ERα by endogenous oestrogen plays a role in the development of PH in SERT^+^ female mice. Curiously, ERα protein expression was actually decreased in the lungs from the SERT^+^ mice. However, we also show that serotonin is a stimulus for ERα expression in hPASMCs. This suggests that the reduction in ERα expression may be due to the reduction in extracellular serotonin concentrations due to the increased SERT activity. However, clearly there is sufficient ERα expression remaining to mediate PH in the SERT^+^ mice. Hypoxia response elements have been identified on the promoters of both ERα and ERβ^28^ and in breast cancer cells, it has been demonstrated that hypoxia induces ESR1 repression at the transcriptional level in a process dependent on hypoxia-inducible factor 1α.^29^ This is consistent with our observation that the ERα antagonist also reversed the increased PH phenotype observed in the hypoxic SERT^+^ mice.

ERβ protein expression was also reduced in the pulmonary arteries of female normoxic SERT^+^ mice. Down-regulation of ERβ has previously been observed in the lung and right heart in a right heart failure rat model.^30^ It has previously been shown that loss of ERβ in female mice leads to abnormal lung structures and systemic hypoxia contributing to ventricular hypertrophy.^31^ ERβ is likely therefore to be protective in experimental PH as previously described in male rodent models.^32,33^ The reduced expression of ERβ we observe in female human PASMCs from PAH patients and in the female SERT^+^ model of PH may result in a loss of the protective effects of oestrogen.

Male mice have lower circulating levels of oestrogen,^5^ and as shown here, low ERα expression. It would not be surprising therefore, if oestrogen plays a lesser role in the development of PH in males. This is consistent with our observation that MPP only prevented the development of hypoxia-induced PH in female mice and not male mice and inhibition of oestrogen synthesis is protective only in female rodent (sugen/hypoxic and hypoxic) models of PH.^5^ As we have demonstrated before, there was no RVH in our normoxic SERT^+^ mice.^16^ Indeed we have demonstrated that normoxic mice are resistant to changes in RVH in the face of moderate changes in pulmonary pressures.^10^ MPP also failed to influence the hypoxia-induced elevation in RVH in the mice. There is evidence that while oestrogen is pathogenic to the pulmonary circulation, it may be protective in the right ventricle. Women are frequently reported to have an improved prognosis compared with men despite their predisposition to developing PAH.^34,35^ This has been attributed to an RV cardioprotective effect of oestrogen. Indeed oestrogen levels correlate with higher right-ventricular ejection fraction and survival in females.^36,37^ Protective effects of exogenous oestrogen in PH observed in other studies may therefore arise from a direct structural effect on the right ventricle and an influence on right-ventricular function as opposed to an effect on vascular remodelling and pulmonary artery pressures. It is possible that the protective influence of oestrogen on the right ventricle may be mediated predominantly by ERβ.^38,39^ We see no effect of MPP on systemic arterial function following treatment with MPP and so the haemodynamic effects of ERα inhibition are selective to the pulmonary circulation. From this study, we suggest oestrogen, via ERα, is implicated in creating a pro-proliferative environment in the pulmonary arteries leading to excessive pulmonary vascular remodelling *in vivo*.

Excessive smooth muscle cell proliferation is a main component of the pulmonary vascular remodelling and vascular lesions observed in PAH. Estrogen is a pro-proliferative factor in pulmonary smooth muscle cells^6,15^ and breast cancer cells.^40^

This suggests that oestrogen may mediate proliferation of hPASMCs and may contribute to PAH pathology. We investigated this further by examining ERα-mediated proliferation in female hPASMCs. We demonstrate for the first time that oestrogen can induce proliferation of hPASMCs via ERα activation. In addition, we show an ERβ agonist, DPN, and a GPER agonist G1, have no effect on proliferation of PASMCs suggesting that ERα is the receptor that mediates estrogen-induced proliferation in hPASMCs. Moreover, we determine the pro-proliferative effect of oestrogen is dependent on activation of downstream PI3K/Akt and ERK/MAPK signalling. ERK and Akt signalling pathways are closely involved in cardiac hypertrophy and pulmonary vascular remodelling and oestrogen has been shown to regulate activation of Akt signalling in right heart failure.^32,41^ Additionally, selective activation of ERα in endothelial cells in aorta increases ERK expression and ERK1/2-mediated cell proliferation. Furthermore activation of PI3K/Akt in human endothelial cells is dependent on ERα but not ERβ.^42,43^ These results provide some insight into the molecular mechanisms by which oestrogen and ERα may mediate pulmonary vascular remodelling during PH and explain the reduction in pulmonary vascular remodelling observed in females following MPP treatment in SERT^+^ mice.

Loss of BMPR2 function mediates proliferation of PASMCs by reducing induction of cell-cycle inhibitors, the Id proteins, particularly Id1 and Id3 in hPASMCs.^44^ A gene–gender relationship for BMPR2 was proposed in a recent study where BMPR2 expression was shown to be decreased in lymphocytes and whole lung from female patients compared with males and BMPR2 was identified as a gene target of ESR1.^45^ It has previously been shown that BMPR2 protein and mRNA expression are decreased following hypoxia in PH.^46,47^ We show here that BMPR2 mRNA expression is decreased in SERT^+^ mice and this is rescued by MPP. BMPR2 protein expression was not decreased in SERT^+^ lung. However, expression levels of BMPR2 protein have been shown to be already relatively low in female mouse lung compared with male. Indeed we show that MPP increased lung BMPR2 expression in both wild-type and SERT^+^ mouse lung. Hence the therapeutic effects of MPP may be associated with increased BMPR2 expression which would exert an anti-proliferative effect. This is consistent with the ability of anastrozole (and hence decreased endogenous oestrogen) to rescue decreased BMPR2 signalling in female mice^5^ and the proposal that BMPR2 signalling is suppressed via ERα binding to the BMPR2 promoter.^45^

In conclusion, development of PH in female SERT^+^ mice is dependent on oestrogen and here we show this is mediated via the ERα receptor which may rescue BMPR2 expression. We also show there is increased ERα expression in hPASMCs from female PAH patients and that serotonin can increase ERα expression in hPASMCs. 17β-estradiol can induce proliferative signalling in hPASMCs. These conclusions suggest causative and co-operative roles for oestrogen and serotonin in the development of pulmonary hypertension in females and are summarized in Supplementary material online, *Figure S3*.

## Supplementary material

Supplementary material is available at *Cardiovascular Research* online.

## Funding

This work is supported by a programme grant from the British Heart Foundation (BHF), UK (RG/11/7/28916 (MacLean), and a BHF project grant RG/11/7/28916 (MacLean). A.W. was supported by a BHF project grant (FS/09/052/28032).

## References

[1] Mcgoon MD Benza RL Escribano-Subias P Jiang X Miller DP Peacock AJ Pepke-Zaba J Pulido T Rich S Rosenkranz S Suissa S Humbert M Pulmonary arterial hypertension: epidemiology and registries. J Am Coll Cardiol 2013;62 (25 Suppl.):D51–D59. 24355642 10.1016/j.jacc.2013.10.023

[2] Shapiro S Traiger GL Turner M Mcgoon MD Wason P Barst RJ Sex differences in the diagnosis, treatment, and outcome of patients with pulmonary arterial hypertension enrolled in the registry to evaluate early and long-term pulmonary arterial hypertension disease management. Chest 2012;141:363–373. 21757572 10.1378/chest.10-3114

[3] Machado RD Eickelberg O Elliott CG Geraci MW Hanaoka M Loyd JE Newman JH Phillips JA III Soubrier F Trembath RC Chung WK Genetics and genomics of pulmonary arterial hypertension. J Am Coll Cardiol 2009;54 (1 Suppl.):S32–S42. 19555857 10.1016/j.jacc.2009.04.015 PMC3725550

[4] Roberts KE Fallon MB Krowka MJ Brown RS Trotter JF Peter I Tighiouart H Knowles JA Rabinowitz D Benza RL Badesch DB Taichman DB Horn EM Zacks S Kaplowitz N Kawut SM Genetic Risk Factors for Portopulmonary Hypertension in Patients with Advanced Liver Disease. Am J Respir Crit Care Med 2009;179:835–842. 19218192 10.1164/rccm.200809-1472OC PMC2675568

[5] Mair KM Wright AF Duggan N Rowlands DJ Hussey MJ Roberts S Fullerton J Nilsen M Loughlin L Thomas M MacLean MR Sex-dependent influence of endogenous estrogen in pulmonary hypertension. Am J Respir Crit Care Med 2014;190:456–467. 24956156 10.1164/rccm.201403-0483OC PMC4214128

[6] White K Dempsie Y Nilsen M Wright AF Loughlin L MacLean MR The serotonin transporter, gender, and 17 beta oestradiol in the development of pulmonary arterial hypertension. Cardiovasc Res 2011;90:373–382. 21177701 10.1093/cvr/cvq408

[7] Long L MacLean MR Jeffery TK Morecroft I Yang XD Rudarakanchana N Southwood M James V Trembath RC Morrell NW Serotonin increases susceptibility to pulmonary hypertension in BMPR2-deficient mice. Circ Res 2006;98:818–827. 16497988 10.1161/01.RES.0000215809.47923.fd

[8] Eddahibi S Humbert M Fadel E Raffestin B Darmon M Capron F Simonneau G Dartevelle P Hamon M Adnot S Serotonin transporter overexpression is responsible for pulmonary artery smooth muscle hyperplasia in primary pulmonary hypertension. J Clin Invest 2001;108:1141–1150. 11602621 10.1172/JCI12805 PMC209526

[9] Dempsie Y MacRitchie NA White K Morecroft I Wright AF Nilsen M Loughlin L Mair KM MacLean MR Dexfenfluramine and the oestrogen-metabolizing enzyme CYP1B1 in the development of pulmonary arterial hypertension. Cardiovasc Res 2013;99:24–34. 23519266 10.1093/cvr/cvt064 PMC3687748

[10] Dempsie Y Nilsen M White K Mair K Loughlin L Ambartsumian N Rabinovitch M MacLean M Development of pulmonary arterial hypertension in mice over-expressing S100A4/Mts1 is specific to females. Respir Res 2011;12:159. 22185646 10.1186/1465-9921-12-159 PMC3276452

[11] White K Loughlin L Maqbool Z Nilsen M McClure J Dempsie Y Baker AH MacLean MR Serotonin transporter, sex, and hypoxia: microarray analysis in the pulmonary arteries of mice identifies genes with relevance to human PAH. Physiol Genomics 2011;43:417–437. 21303932 10.1152/physiolgenomics.00249.2010 PMC3092337

[12] Nilsson S Koehler KF Gustafsson JA Development of subtype-selective oestrogen receptor-based therapeutics. Nat Rev Drug Discov 2011;10:778–792. 21921919 10.1038/nrd3551

[13] Lahm T Albrecht M Fisher AJ Selej M Patel NG Brown JA Justice MJ Brown MB Van DM Trulock KM Dieudonne D Reddy JG Presson RG Petrache I 17 beta-Estradiol Attenuates Hypoxic Pulmonary Hypertension via Estrogen Receptor-mediated Effects. Am J Respir Crit Care Med 2012;185:965–9680. 22383500 10.1164/rccm.201107-1293OC PMC3359941

[14] Keegan A Morecroft I Smillie D Hicks MN MacLean MR Contribution of the 5-HT1B receptor to hypoxia-induced pulmonary hypertension - Converging evidence using 5-HT1B-receptor knockout mice and the 5-HT1B/1D-receptor antagonist GR127935. Circ Res 2001;89:1231–1239. 11739290 10.1161/hh2401.100426

[15] White K Johansen AK Nilsen M Ciuclan L Wallace E Paton L Campbell A Morecroft I Loughlin L McClure JD Thomas M Mair KM MacLean MR Activity of the estrogen-metabolizing enzyme cytochrome P450 1B1 influences the development of pulmonary arterial hypertension/clinical perspective. Circulation 2012;126:1087–1098. 22859684 10.1161/CIRCULATIONAHA.111.062927

[16] MacLean MR Deuchar GA Hicks MN Morecroft I Shen S Sheward J Colston J Loughlin L Nilsen M Dempsie Y Harmar A Overexpression of the 5-hydroxytryptamine transporter gene - Effect on pulmonary hemodynamics and hypoxia-induced pulmonary hypertension. Circulation 2004;109:2150–2155. 15078799 10.1161/01.CIR.0000127375.56172.92

[17] Marino M Galluzzo P Ascenzi P Estrogen signaling multiple pathways to impact gene transcription. Curr Genomics 2006;7:497–508. 18369406 10.2174/138920206779315737 PMC2269003

[18] Mendelsohn ME Karas RH Molecular and cellular basis of cardiovascular gender differences. Science 2005;308:1583–1587. 15947175 10.1126/science.1112062

[19] Meyer MR Haas E Barton M Gender differences of cardiovascular disease: new perspectives for estrogen receptor signaling. Hypertension 2006;47:1019–1026. 16651458 10.1161/01.HYP.0000223064.62762.0b

[20] Jala VR Radde BN Haribabu B Klinge CM Enhanced expression of G-protein coupled estrogen receptor (GPER/GPR30) in lung cancer. BMC Cancer 2012;12:624. 23273253 10.1186/1471-2407-12-624 PMC3557142

[21] Rajkumar R Konishi K Richards TJ Ishizawar DC Wiechert AC Kaminski N Ahmad F Genomewide RNA expression profiling in lung identifies distinct signatures in idiopathic pulmonary arterial hypertension and secondary pulmonary hypertension. Am J Physiol Heart Circ Physiol 2010;298:H1235–H1248. 20081107 10.1152/ajpheart.00254.2009 PMC2853417

[22] Lindberg MK Moverare S Skrtic S Gao H Dahlman-Wright K Gustafsson JA Ohlsson C Estrogen receptor (ER)-beta reduces ERalpha-regulated gene transcription, supporting a “ying yang” relationship between ERalpha and ERbeta in mice. Mol Endocrinol 2003;17:203–208. 12554748 10.1210/me.2002-0206

[23] Ma Y Qiao X Falone AE Reslan OM Sheppard SJ Khalil RA Gender-specific reduction in contraction is associated with increased estrogen receptor expression in single vascular smooth muscle cells of female rat. Cell Physiol Biochem 2010;26:457–470. 20798531 10.1159/000320569 PMC2929922

[24] Matsumoto T Kakami M Kobayashi T Kamata K Gender differences in vascular reactivity to endothelin-1 (1–31) in mesenteric arteries from diabetic mice. Peptides 2008;29:1338–1346. 18486991 10.1016/j.peptides.2008.04.001

[25] Rubanyi GM Freay AD Kauser K Sukovich D Burton G Lubahn DB Couse JF Curtis SW Korach KS Vascular estrogen receptors and endothelium-derived nitric oxide production in the mouse aorta. Gender difference and effect of estrogen receptor gene disruption. J Clin Invest 1997;99:2429–2437. 9153286 10.1172/JCI119426 PMC508083

[26] Massaro D Massaro GD Estrogen regulates pulmonary alveolar formation, loss, and regeneration in mice. Am J Physiol Lung Cell Mol Physiol 2004;287:L1154–L1159. 15298854 10.1152/ajplung.00228.2004

[27] Massaro D Massaro GD Estrogen receptor regulation of pulmonary alveolar dimensions: alveolar sexual dimorphism in mice. Am J Physiol Lung Cell Mol Physiol 2006;290:L866–L870. 16361355 10.1152/ajplung.00396.2005

[28] Wu MH Lu CW Chang FM Tsai SJ Estrogen receptor expression affected by hypoxia inducible factor-1alpha in stromal cells from patients with endometriosis. Taiwan J Obstet Gynecol 2012;51:50–54. 22482968 10.1016/j.tjog.2012.01.010

[29] Ryu K Park C Lee Y Hypoxia-inducible factor 1 alpha represses the transcription of the estrogen receptor alpha gene in human breast cancer cells. Biochem Biophys Res Commun 2011;407:831–836. 21458421 10.1016/j.bbrc.2011.03.119

[30] Matori H Umar S Nadadur RD Sharma S Partow-Navid R Afkhami M Amjedi M Eghbali M Genistein, a soy phytoestrogen, reverses severe pulmonary hypertension and prevents right heart failure in rats. Hypertension 2012;60:425–430. 22753213 10.1161/HYPERTENSIONAHA.112.191445 PMC4252152

[31] Morani A Barros RP Imamov O Hultenby K Arner A Warner M Gustafsson JA Lung dysfunction causes systemic hypoxia in estrogen receptor beta knockout (ERbeta-/-) mice. Proc Natl Acad Sci USA 2006;103:7165–7169. 16636272 10.1073/pnas.0602194103 PMC1459034

[32] Nadadur RD Umar S Wong G Eghbali M Iorga A Matori H Partow-Navid R Eghbali M Reverse right ventricular structural and extracellular matrix remodeling by estrogen in severe pulmonary hypertension. J Appl Physiol 2012;113:149–158. 22628376 10.1152/japplphysiol.01349.2011 PMC3404831

[33] Umar S Iorga A Matori H Nadadur RD Li J Maltese F van der Laarse A Eghbali M Estrogen rescues preexisting severe pulmonary hypertension in rats. Am J Respir Crit Care Med 2011;184:715–723. 21700911 10.1164/rccm.201101-0078OC PMC3208600

[34] Benza RL Miller DP Gomberg-Maitland M Frantz RP Foreman AJ Coffey CS Frost A Barst RJ Badesch DB Elliott CG Liou TG McGoon MD Predicting survival in pulmonary arterial hypertension: insights from the Registry to Evaluate Early and Long-Term Pulmonary Arterial Hypertension Disease Management (REVEAL). Circulation 2010;122:164–172. 20585012 10.1161/CIRCULATIONAHA.109.898122

[35] Humbert M Sitbon O Chaouat A Bertocchi M Habib G Gressin V Yaici A Weitzenblum E Cordier JF Chabot F Dromer C Pison C Reynaud-Gaubert M Haloun A Laurent M Hachulla E Cottin V Degano B Jais X Montani D Souza R Simonneau G Survival in patients with idiopathic, familial, and anorexigen-associated pulmonary arterial hypertension in the modern management era. Circulation 2010;122:156–163. 20585011 10.1161/CIRCULATIONAHA.109.911818

[36] Kawut SM Al-Naamani N Agerstrand C Rosenzweig EB Rowan C Barst RJ Bergmann S Horn EM Determinants of right ventricular ejection fraction in pulmonary arterial hypertension. Chest 2009;135:752–759. 18849396 10.1378/chest.08-1758 PMC2834785

[37] Ventetuolo CE Ouyang P Bluemke DA Tandri H Barr RG Bagiella E Cappola AR Bristow MR Johnson C Kronmal RA Kizer JR Lima JA Kawut SM Sex hormones are associated with right ventricular structure and function: The MESA-right ventricle study. Am J Respir Crit Care Med 2011;183:659–667. 20889903 10.1164/rccm.201007-1027OC PMC3081282

[38] Pedram A Razandi M Lubahn D Liu J Vannan M Levin ER Estrogen inhibits cardiac hypertrophy: role of estrogen receptor-beta to inhibit calcineurin. Endocrinology 2008;149:3361–3369. 18372323 10.1210/en.2008-0133 PMC2453079

[39] Pedram A Razandi M O'Mahony F Lubahn D Levin ER Estrogen receptor-beta prevents cardiac fibrosis. Mol Endocrinol 2010;24:2152–2165. 20810711 10.1210/me.2010-0154 PMC2958752

[40] Pattarozzi A Gatti M Barbieri F Wurth R Porcile C Lunardi G Ratto A Favoni R Bajetto A Ferrari A Florio T 17beta-estradiol promotes breast cancer cell proliferation-inducing stromal cell-derived factor-1-mediated epidermal growth factor receptor transactivation: reversal by gefitinib pretreatment. Mol Pharmacol 2008;73:191–202. 17959712 10.1124/mol.107.039974

[41] Huang H Joseph LC Gurin MI Thorp EB Morrow JP Extracellular signal-regulated kinase activation during cardiac hypertrophy reduces sarcoplasmic/endoplasmic reticulum calcium ATPase 2 (SERCA2) transcription. J Mol Cell Cardiol 2014;75:58–63. 25008120 10.1016/j.yjmcc.2014.06.018 PMC4157950

[42] Chambliss KL Yuhanna IS Mineo C Liu P German Z Sherman TS Mendelsohn ME Anderson RG Shaul PW Estrogen receptor alpha and endothelial nitric oxide synthase are organized into a functional signaling module in caveolae. Circ Res 2000;87:E44–E52. 11090554 10.1161/01.res.87.11.e44

[43] Meyer MR Haas E Prossnitz ER Barton M Non-genomic regulation of vascular cell function and growth by estrogen. Mol Cell Endocrinol 2009;308:9–16. 19549587 10.1016/j.mce.2009.03.009 PMC2780565

[44] Yang J Li X Li Y Southwood M Ye L Long L Al-Lamki RS Morrell NW Id proteins are critical downstream effectors of BMP signaling in human pulmonary arterial smooth muscle cells. Am J Physiol Lung Cell Mol Physiol 2013;305:L312–L321. 23771884 10.1152/ajplung.00054.2013 PMC3891012

[45] Austin E Hamid R Hemnes A Loyd JE Blackwell T Yu C Phillips JA Gaddipati R Gladson S Gu E West J Lane KB BMPR2 expression is suppressed by signaling through the estrogen receptor. Biol Sex Differ 2012;3:6. 22348410 10.1186/2042-6410-3-6 PMC3310853

[46] Long L Crosby A Yang X Southwood M Upton PD Kim DK Morrell NW Altered bone morphogenetic protein and transforming growth factor-beta signaling in rat models of pulmonary hypertension: potential for activin receptor-like kinase-5 inhibition in prevention and progression of disease. Circulation 2009;119:566–576. 19153267 10.1161/CIRCULATIONAHA.108.821504

[47] Takahashi H Goto N Kojima Y Tsuda Y Morio Y Muramatsu M Fukuchi Y Downregulation of type II bone morphogenetic protein receptor in hypoxic pulmonary hypertension. Am J Physiol Lung Cell Mol Physiol 2006;290:L450–L458. 16361357 10.1152/ajplung.00206.2005

